# Population-based validation of a frailty index using electronic regional healthcare records for public health use

**DOI:** 10.1038/s41598-025-18611-9

**Published:** 2025-09-26

**Authors:** Paola Rebora, Giuseppe Occhino, Giuseppe Bellelli, Olivia Leoni, Silvano Casazza, Maria Grazia Valsecchi, Stefania Galimberti, Carlo Alberto Scirè

**Affiliations:** 1 Bicocca Center of Bioinformatics, Biostatistics and Bioimaging (B4 Center), School of Medicine and Surgery, University of Milano-Bicocca, Monza, Italy; 2 Biostatistics and Clinical Epidemiology, Fondazione IRCCS San Gerardo dei Tintori, Monza, Italy; 3 Strategic Regional Agency for Health and Social Care of Apulia (AReSS Puglia), Bari, Italy; 4 School of Medicine and Surgery, University of Milano-Bicocca, Monza, Italy; 5 Acute Geriatric Unit, Fondazione IRCCS San Gerardo dei Tintori, Monza, Italy; 6 Direzione Generale Welfare, Regione Lombardia, Milan, Italy; 7 Fondazione IRCCS San Gerardo dei Tintori, Monza, Italy

**Keywords:** Frailty index, Electronic health records, Mortality, Hospitalizations, Prediction, Diseases, Health care, Medical research, Risk factors

## Abstract

Frailty indices derived from electronic health records offer an efficient approach to identify vulnerable individuals in the general population. We aimed to validate the electronic-regional healthcare database frailty index (e-RHD-FI) in the general adult population, describe its distribution by sex and age, evaluate its predictive validity for mortality, hospitalization and fragility fractures, and assess the performance of frailty cut-off points. We conducted a population-based study of 8,404,004 adult beneficiaries of the Lombardy Regional Health System. The e-RHD-FI was calculated from 40 health deficits using electronic health records from 2008 to 2018. We assessed its distribution, predictive validity for 1-year mortality, hospitalizations and fragility fractures through multi-state analysis and multivariable models evaluating performance across subgroups. The e-RHD-FI distribution was highly asymmetrical (median 0.0125, first-third quartiles 0–0.0375), with 45.8% of adults having no deficits. The index was higher in older adults. Each 0.1-point increment in e-RHD-FI was associated with doubled 1-year mortality risk (HR 2.12, 99% CI 2.11–2.14), 2.5-fold increased hospitalization rate (IRR 2.51, 99% CI 2.49–2.53), and a 55% higher risk of fragility fracture (HR 1.55, 99% CI 1.53–1.57). The AUC for 1-year mortality was 0.883 (99% CI 0.881–0.884). The e-RHD-FI demonstrates strong predictive validity for adverse outcomes in the general population and can effectively identify at-risk individuals for targeted interventions.

**Supplementary Information:**

The online version contains supplementary material available at 10.1038/s41598-025-18611-9.

## Introduction

Frailty is a clinical condition with multifactorial etiology, characterized by reduced strength, endurance, and physiological function, leading to increased vulnerability to external and internal stressors^1,2^. Its assessment provides valuable insights from a public health perspective identifying people requiring increased healthcare resources or who would benefit from comprehensive geriatric evaluation^1,2^.

Multiple studies have shown that frailty effectively detects risk heterogeneity among individuals of the same chronological age^3–7^ including middle-aged subjects^8–10^. Based on this evidence, international guidelines propose frailty as a means to stratify patients according to their risk and to identify those in need of special care^11^.

The frailty index (FI), based on the progressive accumulation of deficits model introduced by Rockwood and colleagues^12^ represents one of the most widely implemented methods for frailty assessment. It quantifies the number of health deficits (clinical signs, symptoms, diseases, and disabilities) that accumulate with aging and correlates strongly with adverse outcomes, such as mortality and hospitalizations in different contexts^8,13^.

Defined as the ratio between the number of deficits identified across multiple domains and a predetermined number of potential deficits, the FI’s methodological simplicity facilitates large-scale application, enabling identification of at-risk individuals without extensive clinical resources. The FI has demonstrated utility in cross-national frailty comparisons^14^ and in frailty assessment using administrative health data^15,16^.

The design and implementation of a FI for community‑dwelling adults, derived from administrative healthcare databases, offers an innovative opportunity to capture population-level complexity and enables robust risk stratification^17,18^.

Given existing evidence, such an FI would have the potential to stratify risk profiles and support a more efficient allocation of healthcare resources, while also enabling proactive public health interventions. Routine data‑based FIs have been shown to be feasible and valid tools for stratifying risk across different populations and settings, though their routine adoption remains limited to few countries^1,2^.

However, constructing an FI from administrative data remains challenging, as it requires adherence to specific methodological standards—such as ensuring that health domains are balanced—that are often not fully represented in these datasets, which mainly capture diagnoses, procedures, and medication use. The development of a country-specific FI would be important to addresses the Italian National Health Service structure and the unique characteristics of regional administrative databases and to provide a framework for standardized frailty assessment that could be replicated across Italian regions with similar administrative data structures, supporting national health planning initiatives.

In Italy, there were few attempts to quantify frailty using administrative data from some regions^19,20^ but critically none of them used the Rockwood approach and followed the standardized criteria recommended by Searle et al.^21^.

We previously developed and validated an electronic-regional healthcare database frailty index (e-RHD-FI) in community-dwellers with SARS-CoV-2 using electronic regional health databases (e-RHD)^22^. The e-RHD-FI was constructed according to standardized criteria recommended by Searle et al.^21^ and validated against both a clinically-derived FI and the Clinical Frailty Scale^23^ using a set of clinical data prospectively collected in a multicentre study of patients hospitalized for COVID-19 ^24^. This preliminary validation demonstrated that e-RHD-FI had good performance in predicting in-hospital and 30-day mortality, risk of hospital admission, and worsening on the WHO clinical scale in COVID-19 patients. As the e-RHD-FI has been built with a rigorous methodology, including a range of systems and domains, among which functional and social dimensions, and was the only frailty index directly compatible with e-RHD of Lombardy we considered it a good candidate to be used in the general adult population.

We aimed to assess the feasibility and validity of applying the e-RHD-FI to the general adult population using electronic-regional healthcare database of Lombardy. Specifically, our objectives were: (1) to apply the e-RHD-FI to the general population and describe its distribution by sex and age; (2) to evaluate its predictive validity with respect to risk of all-cause hospitalization and mortality; and (3) to analyze the predictive value of the frailty index across different subgroups and assess the performance of the frailty cut-off points in the general population. Among the subgroups we examined mortality following acute insults (urgent hospitalization) to evaluate the ability of e-RHD-FI to capture the reduced resilience characteristic of frailty persons.

## Results

### e-RHD-FI distribution

On January 1st, 2019, the Lombardy Regional Health System provided care for a total of 8,404,004 adults, of whom 4,060,083 (48.3%) were male. The median age was 51 years (1st − 3rd quartile 38–66, Table 1). Baseline characteristics stratified by sex are presented in Supplementary Table 2, revealing important sex-specific patterns in frailty distribution.

**Table 1:** Baseline characteristics of the adult beneficiaries of the Lombardy regional health system cohort on January 1st, 2019.

Cohort	January 1st, 2019
*n*	8,404,004
Male sex (%)	4,060,083 (48.3)
Age, years (median [quartiles])	51 [38, 66]
Age ≥ 65 years (%)	2,288,393 (27.2)
Number of deficits (median [quartiles])	0.5 [0, 1.5]
e-RHD-FI (median [quartiles])	0.0125 [0, 0.0375]
e-RHD-FI = 0, n (%)	3,846,242 (45.8)
e-RHD-FI classes (%)	
[0|–0.056)	6,997,206 (83.3)
[0.056|–0.13)	1,119,904 (13.3)
[0.13 |–0.25)	223,477 (2.7)
[0.25 |–1)	63,417 (0.8)

**Fig. 1:**
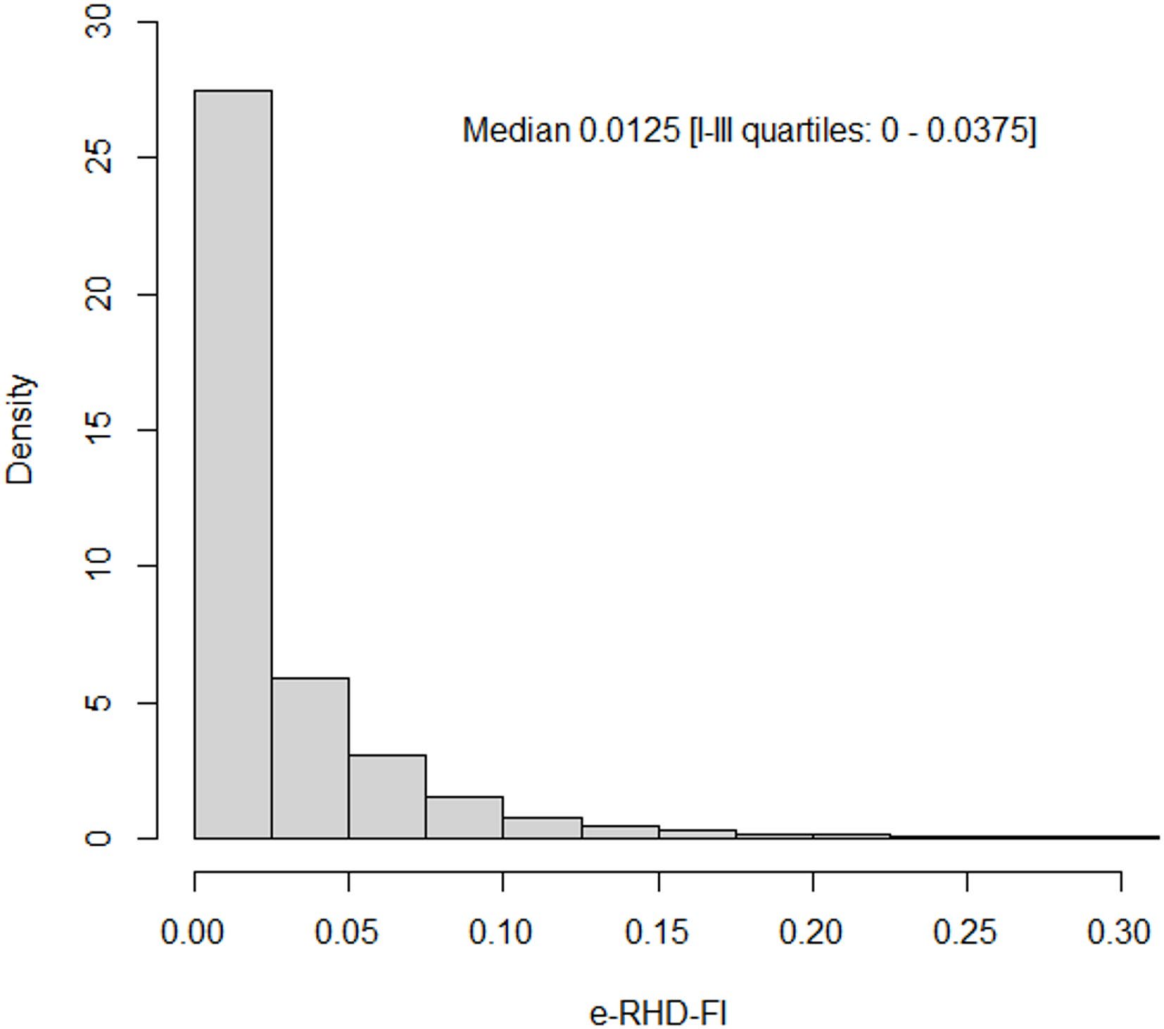
Distribution of e-RHD-FI in adult beneficiaries of the Lombardy Regional Health System cohort on January 1st, 2019.

The distribution of the e-RHD-FI is highly asymmetrical, as depicted in Fig. 1, with a median [quartiles] value of 0.0125 [0, 0.0375]. Almost half of the beneficiaries (3,846,242, 45.8%) had no deficit, with an e-RHD-FI equal to 0, while 3,150,964 (37.5%) had a e-RHD-FI greater than 0 but less than 0.056 (Table 1). A total of 1,119,904 (13.3%) presented an e-RHD-FI between 0.056 and 0.13, 2.7% had values between 0.13 and 0.25 and only 0.8% exceeded 0.25 with these pre-specific cut-offs defined in the methods section. The quartiles of e-RHD-FI were similar in males and females, with the latter having a higher prevalence of frailty mainly due to their older age (Supplementary Table 2); however, as expected, higher values of e-RHD-FI were observed among individuals over 65 years of age. The prevalence of the 40 individual deficits within the cohort is reported in Supplementary Table 1, also differentiated for age class. The most common deficits were hypertension (9.2%), low income (8.3%), trauma (contusion with intact skin surface, 7.9%) and the use of transportation services, including ambulances (7%). Among older adults (≥ 65 years), in addition to those already mentioned above, heart diseases, diabetes and cancer were also common with a prevalence of approximately from 11 to 14%. The geographical distribution of the median value of e-RHD-FI is reported in Supplementary Fig. 1.

### Predictive validity for mortality and hospitalization

**Fig. 2:**
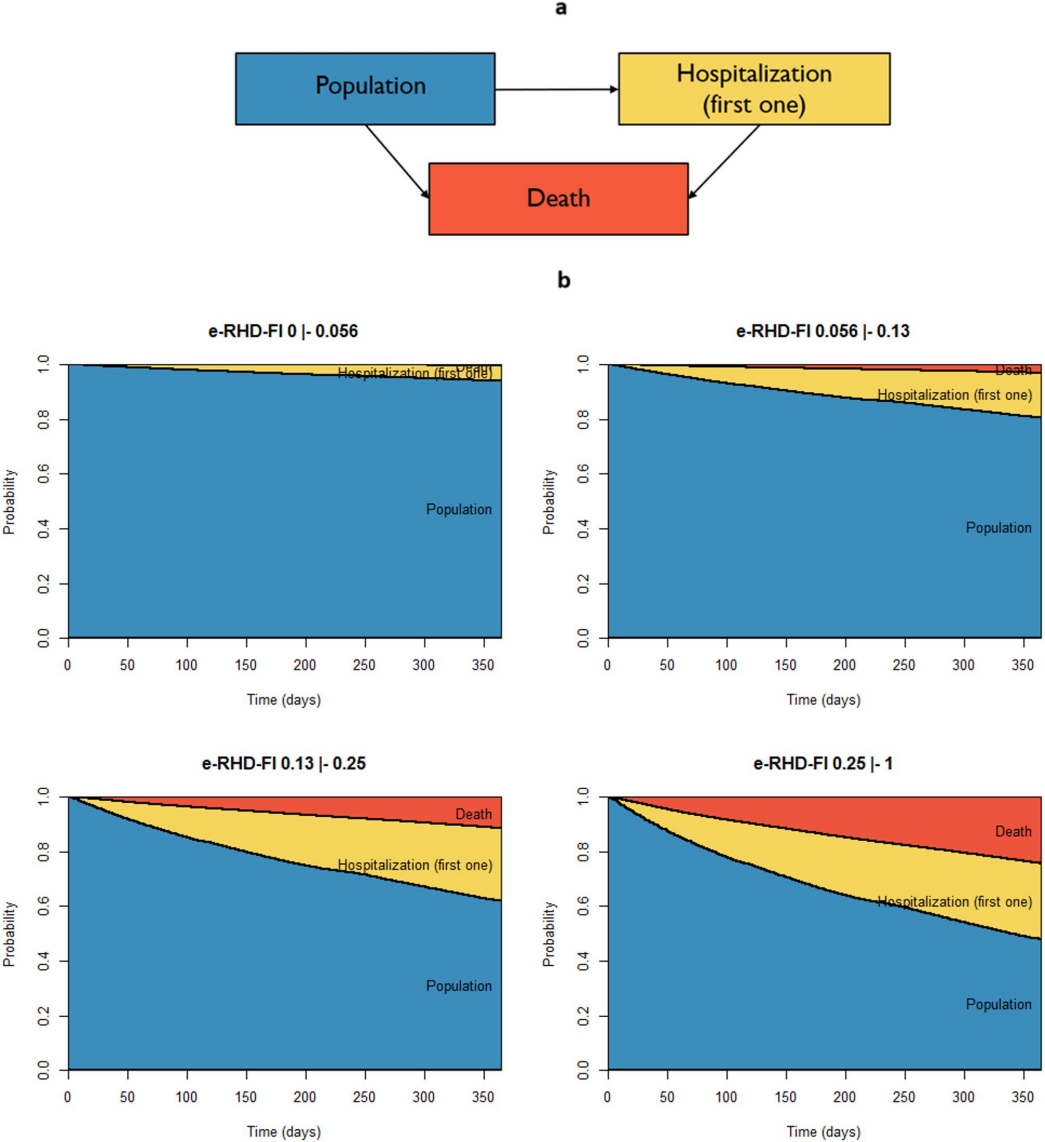
Multi-state model on 1-year health transitions (**a**) and related state occupancy probabilities by e-RHD-FI classes (**b**).

A total of 99,571 (1.2%) deaths and 926,623 hospitalizations affecting 710,419 individuals (8.5% of the population, overall hospitalization rate of 111.4 per 1000 inhabitants) were observed in 2019. Mortality was different according to frailty classes, with rates of 0.3%, 3.2%, 11.8% and 24.8% in individuals with e-RHD-FI below 0.056, between 0.056 and 0.13, between 0.13 and 0.25 and above 0.25, respectively. Figure 2 reports the state occupancy probabilities over the year 2019, as estimated by the multi-state model, which considered a common baseline state for all adults, first hospitalization of the year as a possible transition state, and mortality as the absorbing state, stratified by frailty class. The Figure clearly shows the increased burden of hospitalizations and mortality for increasing levels of e-RHD-FI. In particular, among individuals with an e-RHD-FI ranging from 0.13 to 0.25, 32.2% (71,916/223,477) had at least one hospitalization and 11.8% died (26,310/223,477) during 2019. In the very frail group (e-RHD-FI ≥ 0.25) these percentages raised to 39.6% (25,105/63,417) and 24.8% (15,701/63,417), respectively, resulting in more than half individuals in this category dying or experiencing at least one hospital admission during 2019.

**Fig. 3:**
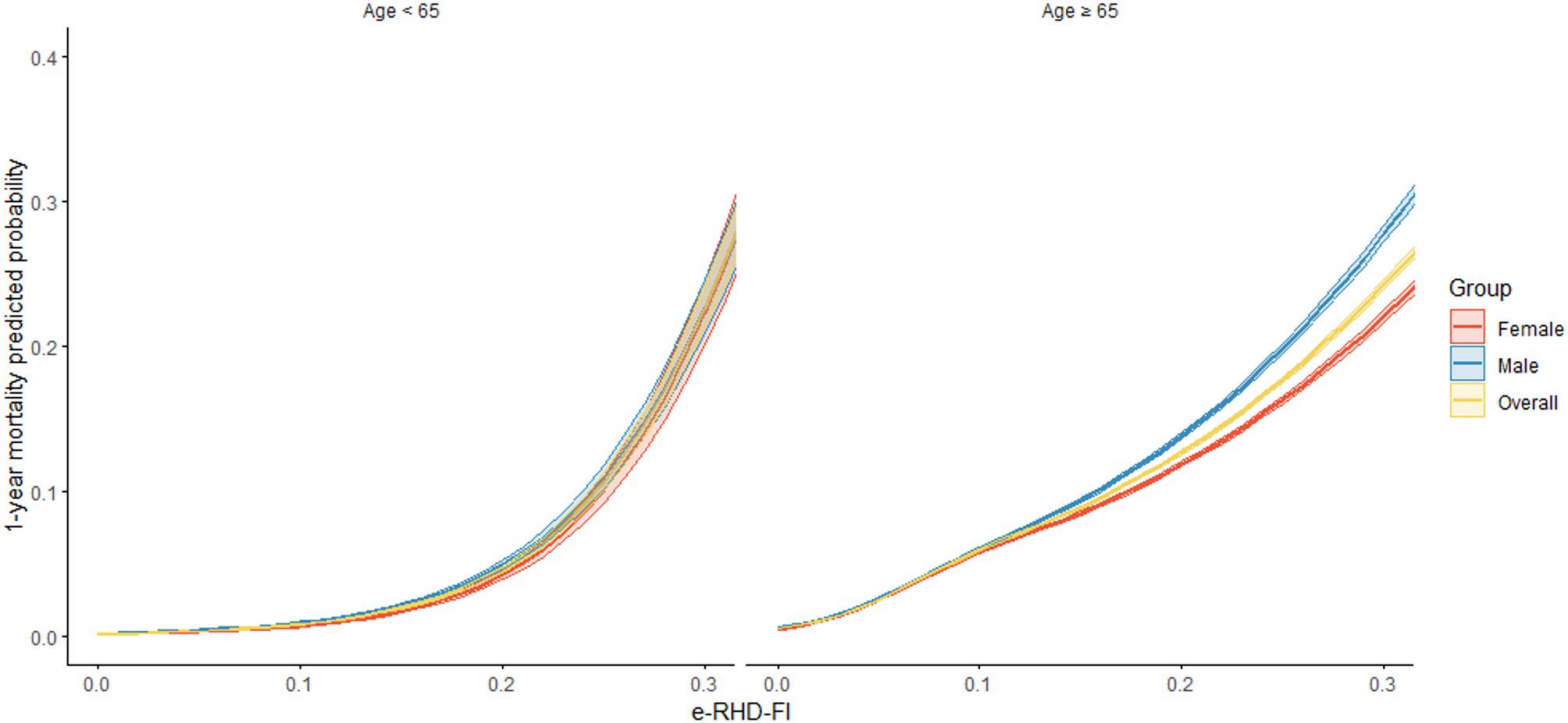
One-year mortality by e-RHD-FI stratified by age and sex based on logistic regression model with restricted cubic spline with three knots placed on 10th, 50th and 90th percentiles.

Mortality by e-RHD-FI, stratified by age and sex, is reported in Fig. 3 (as a continuous variable), and in Supplementary Fig. 2 (by e-RHD-FI classes). In both plots, mortality increased progressively with increasing e-RHD-FI values. Among individuals under 65 years of age, we observed a steeper slope after 0.2 points of e-RHD-FI both in males and females, likely due to the small number (3,824 over 6,115,611) of younger individuals in the very frail class (Fig. 3, left panel). In older adults (Fig. 3, right panel) the relationship between e-RHD-FI and mortality was approximately linear, with males showing higher mortality than females when the e-RHD-FI exceeded 0.1.

**Fig. 4:**
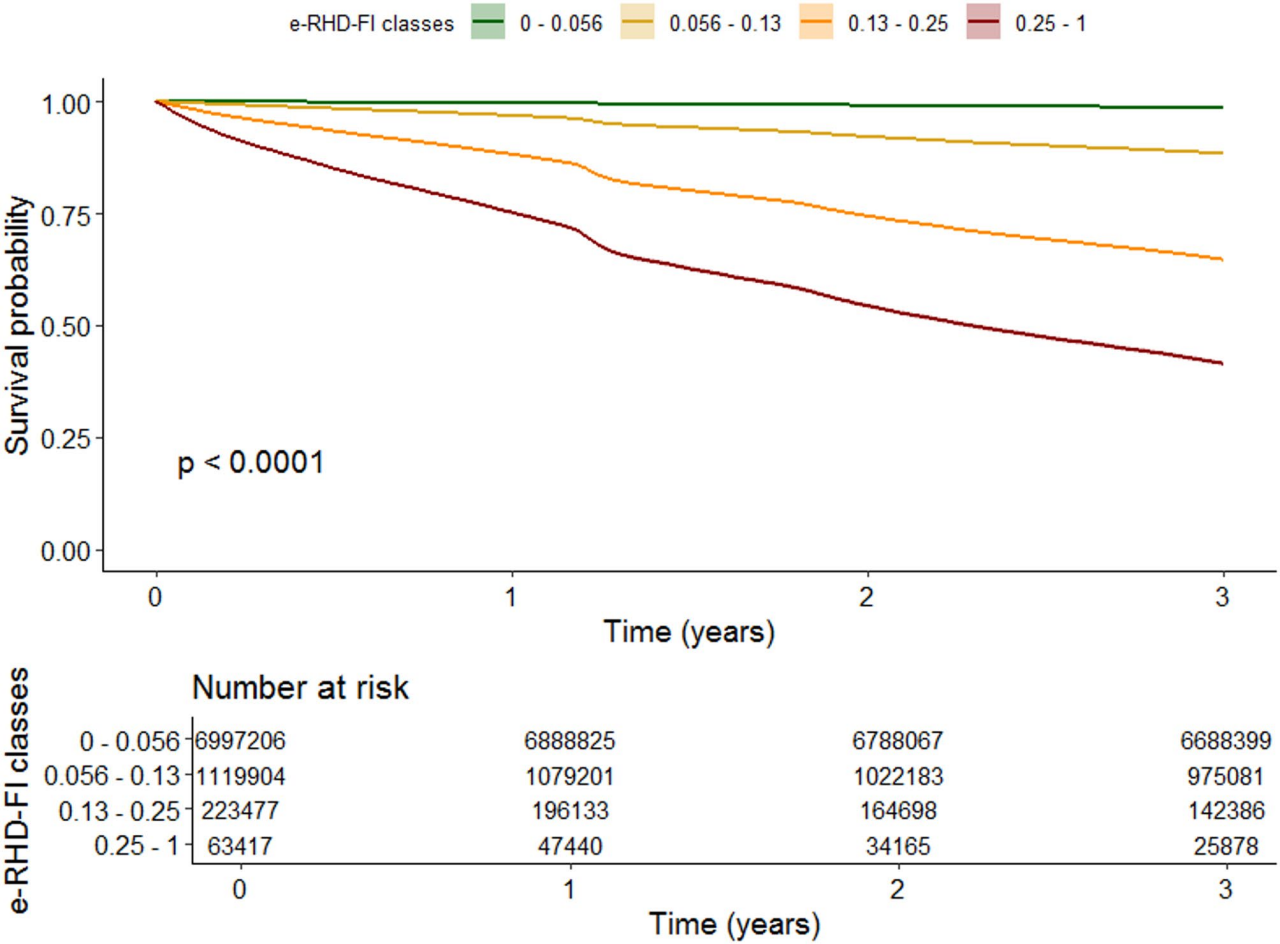
Kaplan Meier curves for 3-year survival across the four e-RHD-FI classes.

Three-year survival estimates by e-RHD-FI classes were reported in Fig. 4. Survival at 3 years was 98.6 (99% CI: 98.6–98.6), 88.3 (99% CI: 88.3–88.4), 64.6 (99% CI: 64.4–64.9), and 41.3 (99% CI: 40.8–41.9) for beneficiaries classified as fit, pre-frail, frail and very frail, respectively, according to their e-RHD-FI status as of January 1st 2019. It is worth noting that a bump in survival is evident in 2020, corresponding to the first wave of SARS-CoV-2 pandemics.

When accounting for sex and age in the multivariable Cox model, each 0.1-point increment of e-RHD-FI was associated with a doubled 1-year mortality hazard (HR: 2.12, 99% CI: 2.11–2.14, Table 2). The hazard ratio of mortality, when stratifying by sex and modeling age and e-RHD-FI flexibly, is depicted in Supplementary Fig. 3.

**Table 2:** Adjusted Cox proportional hazards models and negative binomial regression model results on 1-year mortality, hospitalizations, and fragility fracture, and 30-day mortality after an urgent hospitalization by e-RHD-FI, age and sex. The dispersion parameter for the negative binomial regression on hospitalizations resulted in 0.337.

	1-year outcome			30-day outcome
	Mortality (events = 99,571)	Hospitalizations (events = 926,623)	Fragility fracture (events = 55,626)	Mortality after urgent hospitalization (*N* = 274,629; events = 20,114)
	HR (99% CI)	IRR (99% CI)	HR (99% CI)	HR (99% CI)
e-RHD-FI (0.1-point increment)	2.12 (2.11–2.14)	2.51 (2.49–2.53)	1.55 (1.53–1.57)	1.33 (1.30–1.35)
Age (years)	1.10 (1.10–1.10)	1.02 (1.02–1.02)	1.04 (1.04–1.04)	1.06 (1.06–1.06)
Sex (male vs. female)	1.50 (1.48–1.52)	1.18 (1.18–1.19)	0.62 (0.60–0.63)	1.31 (1.26–1.36)

The rate of hospitalizations increased with e-RHD-FI, with an incidence rate ratio (IRR) of 2.51 (99%CI: 2.49–2.53) each 0.1-point increase, adjusting for age and sex in the negative binomial regression model (Table 2). The dispersion parameter for the model was estimated 0.337.

Over the adult population of Lombardy, 55,626 individual experienced at least a fragility fracture in 2019. When evaluating the association between e-RHD-FI and occurrence of fractures, adjusting for age and sex, we found a HR of 1.55 (99% CI 1.53–1.57, Table 2) for each 0.1-point increment of e-RHD-FI.

In order to assess the predictive ability of e-RHD-FI after an acute insult (resilience) we modeled 30-day mortality in a subpopulation who underwent a hospitalization classified as urgent (*n* = 274,629; events = 20,114) and still found an impact of e-RHD-FI (HR for 0.1-point increment, 1.33, 99% CI: 1.30–1.35, Table 2).

### Predictive performance across subgroups and validation of cut-off points

The AUC of the ROC curve for 1-year mortality (Supplementary Fig. 4, panel a) resulted in 0.883 (99% CI: 0.881–0.884) for e-RHD-FI. The Brier score resulted in 0.011. The cut-off value of 0.056 showed relatively good performance with sensitivity of 78%, specificity of 84% and negative predictive value of 99.7%. The positive predictive value was quite low (5.5%) due to the low incidence of mortality, but was nevertheless higher than the overall mortality rate (1.2%). With increasing cut-offs, as expected, the sensitivity decreased while specificity and positive predictive value increased (Supplementary Fig. 4, panel a).

Focusing on true positives (in our case frail individuals who died within one year) it might be useful to balance the positive predictive value and sensitivity (Supplementary Fig. 4, panel b). Sensitivity was higher at lower thresholds, although precision was low and increased with higher thresholds. The F1 score, i.e. harmonic mean between the positive predictive value and sensitivity, resulted higher (0.22) for the 0.13 cut-off, with a sensitivity of 42% and positive predictive value of 15%. ROC curves for 1-year mortality by sex and age class were reported in Supplementary Fig. 5, with similar values of AUC by sex (panel a). When restricting only to older individuals, AUC resulted in 0.775 (panel b).

## Discussion

In this population-based study, we demonstrated the predictive validity of the electronic-regional healthcare database frailty index (e-RHD-FI) using electronic regional health databases (e-RHD) in the adult population of a region of Italy. Our findings provide robust evidence for the utility of this administrative data-derived frailty index in population health surveillance, clinical decision-making, and research applications.

We successfully applied the e-RHD-FI to over 8.4 million adults in the Lombardy region, demonstrating its feasibility in a general population setting. The distribution analysis revealed that nearly half of the population were fit (e-RHD-FI = 0), while only a small percentage (3.5%) could be classified as frail or very frail according to established cut-offs. As expected, we observed higher FI values among older adults, confirming the age-related nature of frailty accumulation. However, the index detected varying degrees of deficit accumulation across all adult age groups. This distribution is consistent with population-based frailty studies from other developed countries, supporting the external validity of our findings^25,26^.

The e-RHD-FI demonstrated robust predictive validity for both mortality and hospitalization outcomes. Each 0.1-point increment in the index was associated with a doubled risk of 1-year mortality (HR: 2.12) and a 2.5-fold increase in hospitalization rate (IRR: 2.51), even after adjusting for age and sex. These effect sizes are comparable to or exceed those reported for other validated frailty indices, including the original Rockwood FI and international electronic frailty indices.

The multi-state model analysis clearly illustrated how increasing levels of frailty associated with progressively higher probabilities of hospitalization and death, with more than half of very frail individuals experiencing at least one of these outcomes within a year. The e-RHD-FI demonstrated robust predictive validity also for fragility fractures, that represents a critical clinical endpoint, as they reflect the convergence of multiple age-related deficits including reduced bone density, muscle weakness, impaired balance, and increased fall risk, while simultaneously serving as both a consequence of frailty and a catalyst for further functional decline and mortality (HR 1.55).

Our findings validated the previously established cut-off points for frailty classification, with the ROC curve analysis showing good discrimination ability for 1-year mortality (AUC: 0.883). The cut-off of 0.056 demonstrated good sensitivity (78%) and specificity (84%), while the F1 score suggested that 0.13 might represent a good threshold when balancing sensitivity and positive predictive value. The index maintained strong predictive performance across sex subgroups, though with some reduction in discrimination power when restricted to older adults (AUC: 0.775 and Brier Score: 0.034), likely due to the higher baseline risk in this group.

Our findings provide insights into sex differences in frailty prevalence and outcomes, supporting the well-documented sex-frailty paradox described by Arosio and Picca^27^. This paradox describes how women experience greater longevity than men but with higher rates of disability and poor health status, and when frailty is incorporated, women appear frailer with worse health status but remain less susceptible to death than men of the same age. Our results support this paradox in several ways. First, we found higher frailty prevalence in women, particularly in older age groups, consistent with most population studies^15,27^. Furthermore, men over 65 years with the same e-RHD-FI level showed higher mortality rates than women, supporting the “male disadvantage” component of the paradox. This pattern suggests sex-related differences in the rate of deficit accumulation, with men exhibiting a lower tolerance to incremental increases in the Frailty Index compared to women, thereby experiencing a disproportionately higher risk of mortality at equivalent levels of frailty.

Importantly, the e-RHD-FI also demonstrated predictive ability for mortality following acute insults (urgent hospitalizations), supporting its utility in assessing resilience—the capacity to recover from stressors. This finding is consistent with the theoretical understanding of frailty as a state of increased vulnerability and reduced physiological reserve.

Our results align with international experiences where electronic health data-derived frailty indices are increasingly used for public health purposes. For instance, the electronic Frailty Index (eFI) in the UK, developed by Clegg and colleagues^15^is integrated into NHS guidelines to identify frail older adults in primary care and guide geriatric assessments. Similarly, various Claims-Based Frailty Indices (CFIs) in the US, such as the VA-FI and the Kim-CFI, serve as validated tools for research and population health management^28,29^.

The administrative data approach offers significant advantages in scalability and efficiency, enabling continuous, low-cost monitoring of the entire assisted population. As highlighted by Silan et al. in the Italian context, such indicators address the need to stratify the population based on health needs for better intervention planning and resource allocation^19^. Our study confirms the e-RHD-FI’s potential for this purpose in Lombardy. While different CFIs might identify slightly different target populations, their similar predictive performance suggests that using a validated index like the e-RHD-FI provides a robust tool for population-level risk prediction and analysis^30^.

A notable aspect of our study is the e-RHD-FI’s ability to identify and stratify risk across the entire adult population, including middle-aged individuals (< 65 years). While frailty prevalence is significantly higher in older adults, our index also detected deficit accumulation in younger age groups. In fact, mortality risk progressively increased with higher e-RHD-FI values even among those < 65, suggesting frailty’s negative impact begins well before the conventional threshold of 65 years.

This observation is consistent with studies emphasizing a life-course perspective on frailty. In a large UK Biobank study of 493,737 participants aged 37–73 years, 3% of participants were frail and 38% pre-frail, with significant associations between frailty and mortality across all age strata, including middle-aged adults^9^. Similarly, in a Korean prospective cohort from age 53, frailty transitions and burden significantly impact mortality in middle-aged/younger-old adults^31^. A Taiwanese study found that clinically detected frailty predicts unplanned readmissions in patients aged ≥ 50 (17% of the study sample < 65)^32^. Frailty prevalence in younger and middle-aged adults is increasingly recognized, though variable depending on definition and population^10^. These studies highlight that vulnerability associated with frailty is not exclusive to advanced age.

Using administrative data derived instruments like the e-RHD-FI allows for early identification of individuals showing deficit accumulation before age 65. While identifying frailty might present greater challenges in younger populations using administrative data, our study indicates risk signals can be detected^33^. Early identification of frailty trajectories in middle age could have significant implications in prevention. Targeted interventions implemented at this stage could potentially modify the aging trajectory, slowing or reversing progression towards severe frailty and reducing future disability and healthcare burden. The e-RHD-FI, applied routinely, could act as an early warning system, flagging middle-aged individuals at risk for further assessment and personalized preventive interventions.

Beyond public health applications, the validation of e-RHD-FI offers significant implications for pharmacoepidemiology. Studies using large administrative health databases often face ‘confounding by frailty’, especially when examining drug effects in older or multimorbid populations. Frailty, as a state of increased vulnerability, influences both treatment propensity and outcome risk, acting as a strong confounder. Inadequate adjustment for frailty can bias estimates of drug effectiveness and safety^34^.

The e-RHD-FI, developed and validated using the same regional administrative data frequently used for pharmacoepidemiologic studies in Lombardy, represents a particularly suitable covariate. Its strong association with mortality and hospitalization confirms its validity as a summary measure of health status and vulnerability. Incorporating the e-RHD-FI (preferably as a continuous variable or using meaningful thresholds) into pharmacoepidemiologic models can enable more effective control of confounding by frailty, leading to more accurate treatment effect estimates. A preliminary study on the cardiovascular safety of targeted therapies in rheumatoid arthritis in Lombardy already included e-RHD-FI as a covariate and possible effect modifier^35^.

The use of electronic health databases for estimating frailty has several limitations. First, these databases primarily focus on administrative issues and lack comprehensive clinical and social information needed to directly assess all frailty domains. While we included deficits covering various domains, including proxies for socioeconomic status and functional impairments often missing in electronic FIs, capturing aspects like physical performance, psychological well-being, and social factors remains challenging. This was evident in Fig. 3, where at the same level of frailty, males over 65 years of age showed higher mortality compared to females, likely due to socio-demographic, occupational, and lifestyle factors associated with male sex that are not captured by the index, also beyond the sex-frailty paradox. Second, information might be inaccurate or missing due to coding errors, inconsistent reporting, or use of private healthcare, although Italy’s universal health system mitigates the latter concern. Our previous validation found administrative data deficits are sensitive but can lack specificity, potentially leading to misclassification^22^. Third, the index might overlook frail individuals who rarely seek medical attention. This could particularly relevant for some ethnics groups^36–38^ but unfortunately ethnicity was not available in our data and thus we were not able to assess it. Finally, we measured frailty at an index date (January 1st, 2019) and did not consider changes over time in this specific analysis, although the data source allows for longitudinal assessment. The index date allowed us to evaluate 1-year endpoints without COVID-19 influence, but not the longer-term 3-year mortality endpoint. We used calendar time as the time-scale partially accounting for this limitation and found that survival estimates across frailty classes maintained their divergent trajectories during the COVID-19 period (after the first year of observation). Longer-term mortality outcomes warrant further evaluation in more recent years.

Despite these limitations, this population-based study demonstrates that the e-RHD-FI, derived from regional electronic health databases, is a feasible and valid tool for assessing frailty in the general adult population of Lombardy. It shows strong predictive validity for mortality and hospitalization, performs well across different subgroups, and corresponds with established frailty cut-offs. The e-RHD-FI offers valuable insights on a large scale and can identify subjects for more comprehensive clinical assessments or targeted public health interventions. Furthermore, it represents a valuable resource for improving the validity of pharmacoepidemiologic research by enabling better control for confounding by frailty. Its application supports evidence-based health planning and preventive strategies aimed at mitigating the impact of frailty on individuals and the healthcare system.

## Methods

### Data source and population

Adult beneficiaries of the Lombardy Regional Health System are our target population. We retrieved adult beneficiaries on January 1st, 2019 by the Population Register (Demographics NAR) and we followed them up to death, emigration/end of assistance in the region or last available follow-up time (December 31st, 2021), whichever occurs first.

The study was approved by the Ethical Committee of the University of Milano-Bicocca (approval number 920, protocol n. 0065413 dated 03/02/2025-UOR:003406), which also waived the requirement to obtain informed consent. All methods were performed in accordance with relevant guidelines and regulations.

### Electronic-regional healthcare database frailty index (e-RHD-FI)

The electronic-regional healthcare database frailty index (e-RHD-FI) previously developed^22^ was applied considering the dates of January 1st, 2019 as the index date.

The e-RHD-FI was developed, according to Rockwood’s approach, with 40-items/deficits and considering a recall period from 1 to 10 years, according to different types of health deficits and determinants. The e-RHD-FI was calculated as the ratio between the sum of health deficits and determinants and the total number of health deficits and determinants included in the index (theoretical range 0–1). For each patient, all deficits contributed to the index with 1 point, except income, which was classified into three categories: upper-middle/high income (no contribution to the index), lower-middle income (0.5 contribution), and low income (1 point contribution). Health deficits and determinants were retrieved by cross-referencing the different data sources in the e-RHD. Health deficits were defined as symptoms, signs, disabilities, and diseases, covering a range of systems and domains (i.e., physical, mental and social). The co-payment exemptions database, Integrated Home Assistance, and Intermediate Assistance Observation Sheet datasets were evaluated to obtain a proxy for socio-economic status and to identify nursing home residents. Physical disabilities were retrieved from exemptions, Integrated Home Assistance, and prosthesis delivery service. Mental illness data were used to assess the mental domain^19,39^. Full details on data source and algorithm development are reported elsewhere^22^.

### End-points

The primary end-point is 1-year mortality (during the year 2019 in our context). We will also consider the number of hospitalizations occurring in 1 year, a combined end-point of death and hospitalizations and the occurrence of fractures typically due to fragility (International Classification of Diseases, 9th Revision, Clinical Modification, ICD9-CM, codes 733.1*; 820*-821*; 805*; 813*-814*). Finally, we also considered a longer-term mortality end-point (3 years).

Mortality was retrieved by the Population Register, while for hospitalizations we used the Hospital Discharge Form. We considered all hospitalizations except for those due to delivery (DRG codes between 370 and 391).

### Statistical methods

The e-RHD-FI was computed on adult beneficiaries of the Lombardy Regional Health System on January 1st, 2019 by using electronic-regional healthcare databases from 2008 up to 2018. Variables included in the e-RHD-FI, along with their prevalence, were reported in the overall population as well as for adults younger than 65 years and those aged 65 years or older. The resulting e-RHD-FI was described by median and quartiles and represented by histogram. Previously identified/used cut-offs were used to classify adults as fit (< 0.056 ^22^), pre-frail (0.056–0.13), frail (0.13–0.25) and very frail (≥ 0.25). The median value of e-RHD-FI was computed also across different territories of Health Protection Agencies in Lombardy and reported in a map to depict the geographical distribution. Proportions of 1-year mortality and first hospitalization were graphically represented by e-RHD-FI (< 0.056, 0.056–0.13, 0.13–0.25 or ≥ 0.25), age (< 65 or ≥ 65 years) and sex classes.

To describe hospitalizations and mortality at different levels of frailty we used a multi-state (illness-death model) model with three states: (1) a baseline state event-free common to all adults, (2) first hospitalization and (3) mortality as an absorbing state. We accounted for age and sex and stratified the model on frailty class.

The relationship between e-RHD-FI and 1-year mortality was modeled by a logistic regression model with a restricted cubic spline with three knots placed on 10th, 50th and 90th percentiles, respectively, and stratifying by age (< 65 vs. ≥ 65 years) and sex. Survival up to three years was estimated using the Kaplan–Meier method and compared across frailty classes using the log-rank test. The associations between the e-RHD-FI and 1-year mortality and fragility fractures were evaluated by Cox proportional hazards regression models, adjusting by continuous age and stratifying by sex. Restricted cubic splines with three knots have been used to relax the linearity in the relationship between the e-RHD-FI and the logarithm of the mortality hazard. The Brier score was computed in the overall population, as well as for adults younger than 65 years and those aged 65 years or older.

The number of hospitalizations occurring in 2019 was also derived, and rates of hospitalizations were computed at different levels of e-RHD-FI and compared by a negative binomial regression model including sex and continuous age with the length of follow-up used as offset. Furthermore, we restricted the population to those who underwent an urgent hospitalization during 2019, and evaluated the association between e-RHD-FI and 30-day mortality after that hospital admission by a Cox proportional hazards regression model adjusting by sex and age.

The performance of the frailty index in predicting 1-year mortality was assessed by the area under the Receiver-Operating-Characteristic (ROC) curve (AUC) and the Precision-Recall curve. The performance of the three cut-offs previously used were reported by specific values of sensibility *(also called recall)*, specificity, Positive Predictive Value (PPV, *also called precision*), and Negative Predictive Value (NPV). For each cut-off the harmonic mean between precision and recall was reported (F1 score). Furthermore, AUC values were also computed stratifying the population according to sex and age (< 65 vs. ≥ 65 years).

## Supplementary Information

Below is the link to the electronic supplementary material.

Supplementary Material 1

## Data Availability

The data that support the findings of this study are available from Regione Lombardia but restrictions apply to the availability of these data, which were used under license for the current study, and so are not publicly available. Data are however available from the corresponding author upon reasonable request and with permission of Regione Lombardia.
