# Supplementary material for: Population-based validation of a frailty index using electronic regional healthcare records for public health use
Source: Sci Rep. 2025 Sep 26;15:33298. doi: 10.1038/s41598-025-18611-9 (PMC12475065; doi:10.1038/s41598-025-18611-9)

# Supplementary material

**Supplementary Table 1:** Variables included in the electronic Regional Healthcare Database Frailty Index (e-RHD-FI) and their prevalence in adult beneficiaries of the Lombardy Regional Health System cohorts on January 1st, 2019

|  | **Cohort** | **January 1st, 2019** | **Age < 65 years** | **Age ≥ 65 years** |
| --- | --- | --- | --- | --- |
| **Defict ID** | **n** | **8404004** | **6115611** | **2288393** |
| FC_001 | Respiratory failure/Oxygen therapy (n, %) | 5942 (0.1) | 588 (0.0) | 5354 (0.2) |
| FC_009 | Arthritis and related disorders (n, %) | 441301 (5.3) | 238358 (3.9) | 202943 (8.9) |
| FC_012 | Cerebrovascular disease (n, %) | 243694 (2.9) | 75116 (1.2) | 168578 (7.4) |
| FC_014 | Chronic obstructive pulmonary disease and allied conditions (n, %) | 295864 (3.5) | 129827 (2.1) | 166037 (7.3) |
| FC_017 | Contusion with intact skin surface (n, %) | 665122 (7.9) | 481283 (7.9) | 183839 (8.0) |
| FC_019 | Diabetic foot (n, %) | 7277 (0.1) | 840 (0.0) | 6437 (0.3) |
| FC_024 | Diseases of endocrine glands (including diabetes) (n, %) | 218383 (2.6) | 53175 (0.9) | 165208 (7.2) |
| FC_025 | Venous vascular disease (n, %) | 33700 (0.4) | 13484 (0.2) | 20216 (0.9) |
| FC_032 | Hereditary and degenerative diseases of the central nervous system (n, %) | 66725 (0.8) | 11528 (0.2) | 55197 (2.4) |
| FC_034 | Special bed provided by the Health Regional System (n, %) | 18640 (0.2) | 1130 (0.0) | 17510 (0.8) |
| FC_037 | Hypertension (n, %) | 1836261 (21.8) | 560164 (9.2) | 1276097 (55.8) |
| FC_038 | Ill-defined and unknown causes of morbidity and mortality (n, %) | 125498 (1.5) | 78763 (1.3) | 46735 (2.0) |
| FC_043 | Ischemic myocardial disease (n, %) | 246215 (2.9) | 52401 (0.9) | 193814 (8.5) |
| FC_050 | Chronic kidney disease (n, %) | 69426 (0.8) | 10783 (0.2) | 58643 (2.6) |
| FC_051 | Neurotic disorders, personality disorders, and other nonpsychotic mental disorders (n, %) | 82121 (1.0) | 50899 (0.8) | 31222 (1.4) |
| FC_053 | Nurse care provided at home (n, %) | 79960 (1.0) | 7867 (0.1) | 72093 (3.2) |
| FC_057 | Open wound of the lower limb (n, %) | 58082 (0.7) | 24321 (0.4) | 33761 (1.5) |
| FC_058 | Organic psychotic conditions (n, %) | 60562 (0.7) | 5366 (0.1) | 55196 (2.4) |
| FC_062 | Bacterial diseases (n, %) | 91437 (1.1) | 28045 (0.5) | 63392 (2.8) |
| FC_064 | Diseases of the urinary system (n, %) | 311849 (3.7) | 159355 (2.6) | 152494 (6.7) |
| FC_066 | Heart diseases (n, %) | 407988 (4.9) | 91389 (1.5) | 316599 (13.8) |
| FC_069 | Psychosis (n, %) | 54514 (0.6) | 33914 (0.6) | 20600 (0.9) |
| FC_070 | Diabetes supplies (n, %) | 363025 (4.3) | 111996 (1.8) | 251029 (11.0) |
| FC_077 | Pneumonia and influenza (n, %) | 169312 (2.0) | 71594 (1.2) | 97718 (4.3) |
| FC_090 | Transportation services including ambulance (n, %) | 712752 (8.5) | 429136 (7.0) | 283616 (12.4) |
| FC_092 | Walking aids and attachments (n, %) | 25991 (0.3) | 2348 (0.0) | 23643 (1.0) |
| FC_093 | Wheelchairs (n, %) | 16700 (0.2) | 2794 (0.0) | 13906 (0.6) |
| FC_100 | Osteoporosis (fragility fractures) (n, %) | 215262 (2.6) | 89346 (1.5) | 125916 (5.5) |
| FC_114 | Hearing impairment (n, %) | 28447 (0.3) | 5986 (0.1) | 22461 (1.0) |
| FC_115 | Visual impairment (n, %) | 1387 (0.0) | 534 (0.0) | 853 (0.0) |
| FC_118 | Cancer (n, %) | 439801 (5.2) | 166947 (2.7) | 272854 (11.9) |
| FC_125 | Dependency in self-care (n, %) | 92849 (1.1) | 8892 (0.1) | 83957 (3.7) |
| FC_126 | Dependency in self-dressing (n, %) | 92630 (1.1) | 8875 (0.1) | 83755 (3.7) |
| FC_127 | Dependency in walking (n, %) | 97308 (1.2) | 9255 (0.2) | 88053 (3.8) |
| FC_128 | Dependency in toileting (n, %) | 85196 (1.0) | 7907 (0.1) | 77289 (3.4) |
| FC_129 | Dependency in self feeding (n, %) | 67298 (0.8) | 6378 (0.1) | 60920 (2.7) |
| FC_133 | Living in a nursing home (n, %) | 19902 (0.2) | 433 (0.0) | 19469 (0.9) |
| FC_134 | Living alone (n, %) | 2493 (0.0) | 113 (0.0) | 2380 (0.1) |
| FC_135 | Income (n, %) | |  |  |
|  | Upper-middle and high income | 5447043 (64.8) | 5004008 (81.8) | 443035 (19.4) |
|  | Lower-middle income | 2213841 (26.3) | 603968 (9.9) | 1609873 (70.3) |
|  | Low income | 743120 (8.8) | 507635 (8.3) | 235485 (10.3) |
| FC_137 | End-stage renal disease (hemodialysis) (n, %) | 8359 (0.1) | 2892 (0.0) | 5467 (0.2) |
|  | **1-year death (n, %)** | **99571 (1.2)** | **10366 (0.2)** | **89205 (3.9)** |
|  | **1-year end of assistance (n, %)** | **92355 (1.1)** | **83123 (1.4)** | **9232 (0.4)** |
|  | **1-year still assisted on Dec 31^st^, 2019 (n, %)** | **8212078 (97.7)** | **6022122 (98.5)** | **2189956 (95.7)** |

**Supplementary Table 2:** Baseline characteristics of the adult beneficiaries of the Lombardy Regional Health System cohort on January 1^st^, 2019 stratified by sex

| **Cohort** |  | **Males** | **Females** |
| --- | --- | --- | --- |
| **n** |  | **4,060,083 (48.3)** | **4,343,921 (51.7)** |
| **Age, years (median [quartiles])** |  | 50 [37, 64] | 52 [39, 68] |
| **Age ≥ 65 years (%)** |  | 986,646 (24.3) | 1,301,747 (30.0) |
| **Number of deficits (median [quartiles])** |  | 0.5 [0, 1.5] | 0.5 [0, 1.5] |
| **e–RHD–FI (median [quartiles])** |  | 0.0125 [0, 0.0375] | 0.0125 [0, 0.0375] |
| **e–RHD–FI = 0, n (%)** |  | 1,928,192 (47.5) | 1,918,050 (44.2) |
| **e–RHD–FI classes (%)** |  |  |  |
| **[0 \|– 0.056)** |  | 3,395,101 (83.6) | 3,602,105 (82.9) |
| **[0.056 \| – 0.13)** |  | 533,769 (13.1) | 586,135 (13.5) |
| **[0.13 \| – 0.25)** |  | 106,361 (2.6) | 117,116 (2.7) |
| **[0.25 \|– 1)** |  | 24,852 (0.6) | 38,565 (0.9) |

**Supplementary Figure 1:** Geographical distribution of the median value of e-RHD-FI value across different territories of Health Protection Agencies in Lombardy. Data are displayed by Provinces, some Health Protection Agencies includes more provinces (i.e. ATS Città Metropolitana di Milano covers Milan metropolitan area, Milan and Lodi, ATS Brianza (covering parts of Monza-Brianza and Lecco provinces), ATS Insubria (covering Como and Varese provinces), ATS Val Padana (covering Cremona and Mantova provinces)). People with the residence in a nursing home are not assigned to a specific territory.


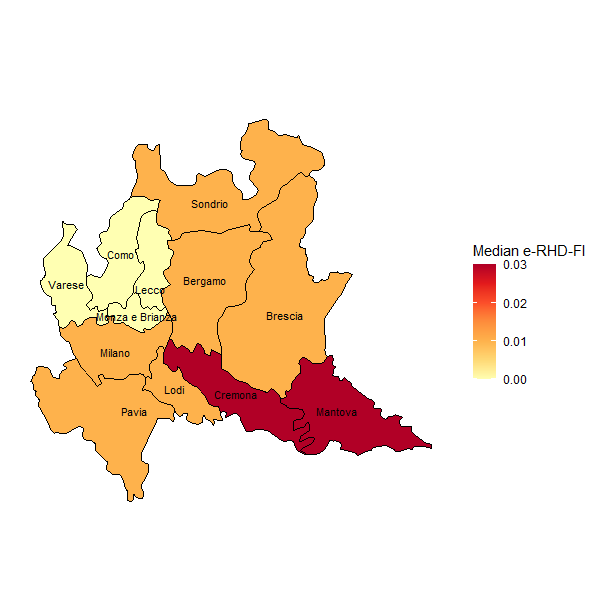


**Supplementary Figure 2:** Proportions of one-year mortality and hospitalization by e-RHD-FI, age and sex classes

**
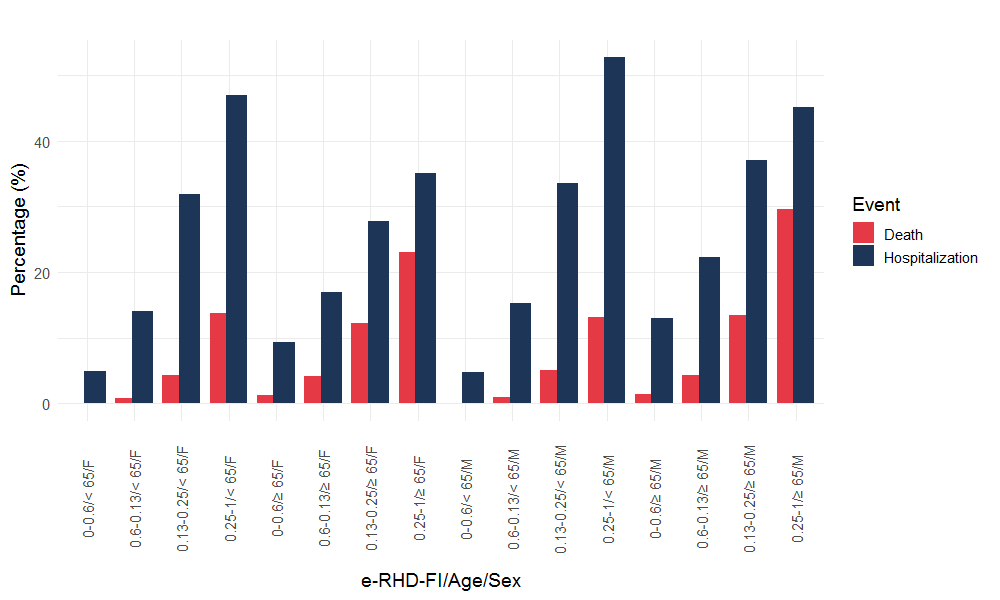
**

**Supplementary Figure 3:** Hazard Ratios (HR) on 1-year mortality of e-RHD-FI with restricted cubic spline with three knots placed on 10^th^, 50^th^ and 90^th^ percentiles by Cox proportional hazards models adjusted for age and stratified by sex.


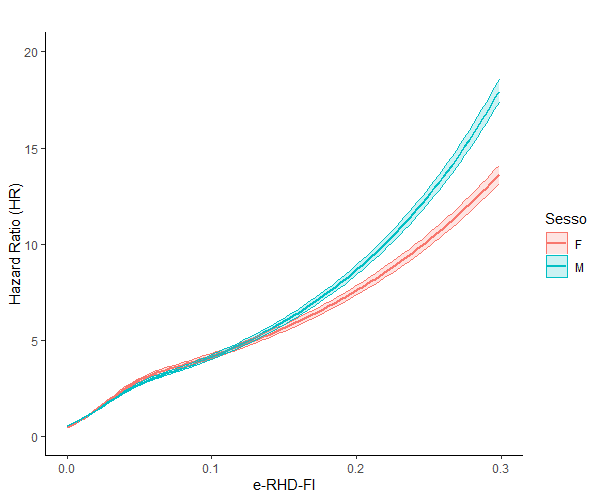


**Supplementary Figure 4:** Receiver-operating characteristic (a) and Positive predictive value and sensitivity (Precision-Recall) (b) curves on 1-year mortality by e-RHD-FI with performance metrics for the three cut-offs.

1. Receiver-operating characteristic curve reports the sensitivity and specificity values on 1-year mortality according to different possible cut-offs of e-RHD-FI. The performance of the three cut-offs previously used are reported with red dots and specific values of Se, Sp, PPV, NPV are reported.
2. In machine learning literature the curve in (b) is called precision (PPV) recall (Se) curve, where precision is the positive predictive value and recall the sensitivity. For each cut-off the harmonic mean between Positive predictive value and sensitivity is reported (F1 score). The performance of the three cut-offs previously used are reported with red dots and specific values of Se-recall, PPV-precision, and F1 score are reported.


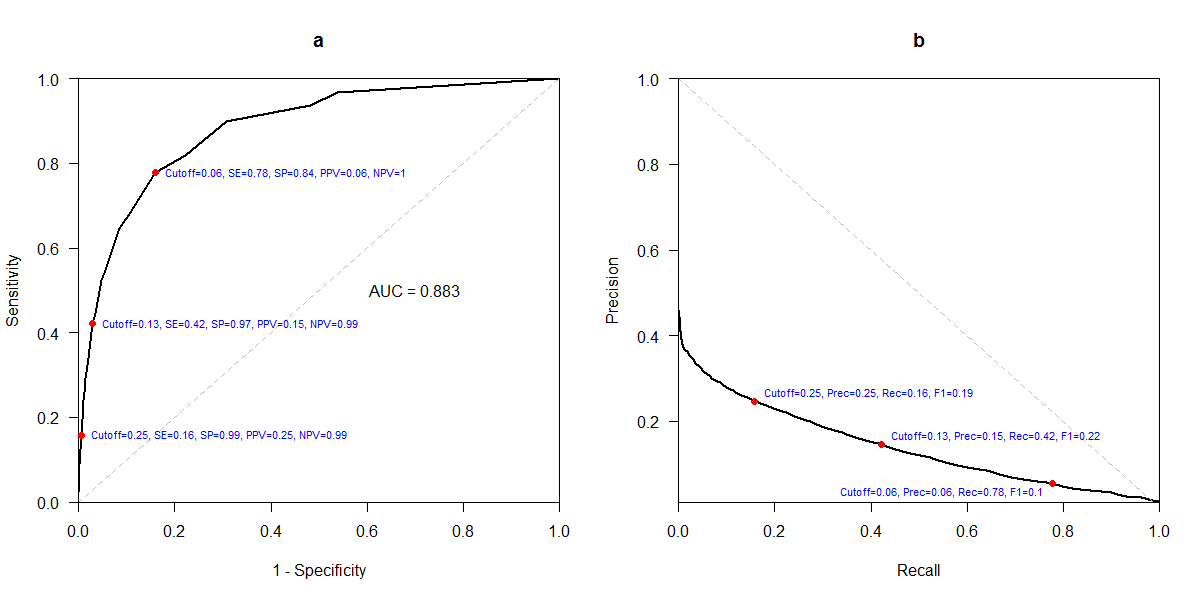


**Supplementary Figure 5:** Receiver-operating characteristic (ROC) curves on 1-year mortality by e-RHD-FI by sex (panel a) and age (panel b)


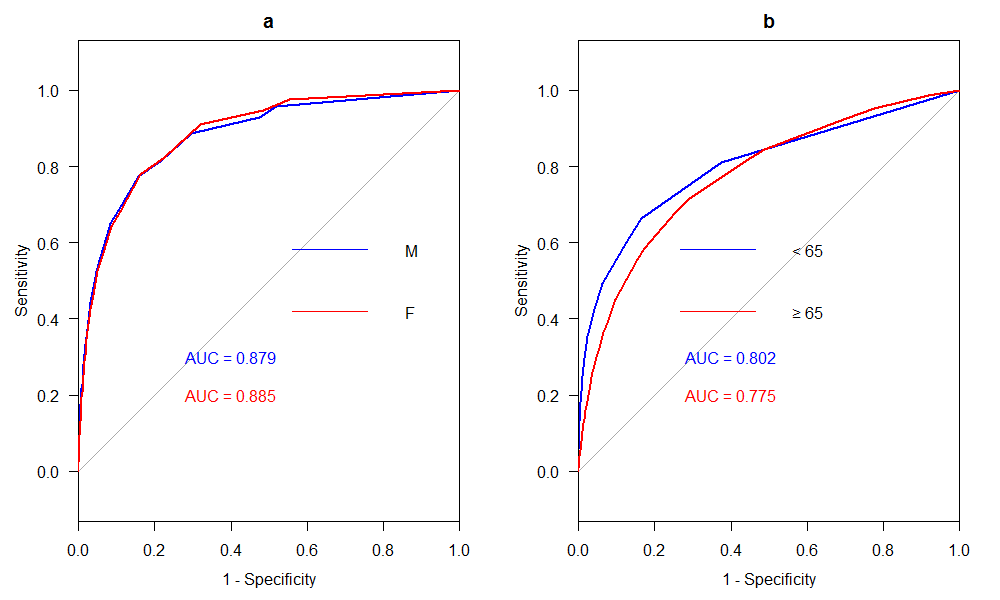

Supplement: Supplementary file 1 — Supplementary Material 1 [file 41598_2025_18611_MOESM1_ESM.docx]
